# Heritability of fat distributions in male mice from the founder strains of the Diversity Outbred mouse population

**DOI:** 10.1093/g3journal/jkab079

**Published:** 2021-03-15

**Authors:** Brendan T Keenan, Jeanette C Webster, Andrew S Wiemken, Nir Lavi-Romer, Teresa Nguyen, Karen L Svenson, Raymond J Galante, Gary A Churchill, Stephen Pickup, Allan I Pack, Richard J Schwab

**Affiliations:** 1 Division of Sleep Medicine, Department of Medicine, University of Pennsylvania, Philadelphia, PA 19104, USA; 2 The Jackson Laboratory, Bar Harbor, ME 04609, USA; 3 Department of Radiology, University of Pennsylvania, Philadelphia, PA 19104, USA

**Keywords:** mice, Diversity Outbred, Collaborative Cross, founder strains, fat distributions, magnetic resonance imaging, heritability

## Abstract

Specific fat distributions are risk factors for complex diseases, including coronary heart disease and obstructive sleep apnea. To demonstrate the utility of high-diversity mouse models for elucidating genetic associations, we describe the phenotyping and heritability of fat distributions within the five classical inbred and three wild-derived founder mouse strains of the Collaborative Cross and Diversity Outbred mice. Measurements of subcutaneous and internal fat volumes in the abdomen, thorax and neck, and fat volumes in the tongue and pericardium were obtained using magnetic resonance imaging in male mice from the A/J (*n* = 12), C57BL/6J (*n* = 17), 129S1/SvlmJ (*n* = 12), NOD/LtJ (*n* = 14), NZO/HILtJ (*n* = 12), CAST/EiJ (*n* = 14), PWK/PhJ (*n* = 12), and WSB/EiJ (*n* = 15) strains. Phenotypes were compared across strains using analysis of variance and heritability estimated as the proportion of phenotypic variability attributable to strain. Heritability ranged from 44 to 91% across traits, including >70% heritability of tongue fat. A majority of heritability estimates remained significant controlling for body weight, suggesting genetic influences independent of general obesity. Principal components analysis supports genetic influences on overall obesity and specific to increased pericardial and intra-neck fat. Thus, among the founder strains of the Collaborative Cross and Diversity Outbred mice, we observed significant heritability of subcutaneous and internal fat volumes in the neck, thorax and abdomen, pericardial fat volume and tongue fat volume, consistent with genetic architecture playing an important role in explaining trait variability. Findings pave the way for studies utilizing high-diversity mouse models to identify genes affecting fat distributions and, in turn, influencing risk for associated complex disorders.

## Introduction

Obesity is a growing epidemic with numerous public health consequences, including hypertension, myocardial infarction, stroke, cardiac arrhythmias, and obstructive sleep apnea (OSA) (Bixler *et al.* 2005; Van Gaal *et al.* 2006; Punjabi 2008; Landsberg *et al.* 2013; Lim and Pack 2017). However, general measures of obesity, including body weight or body mass index (BMI), may not best capture the obesity-related risk for these outcomes (Cornier *et al.* 2011). Certain distributions of fat, including visceral and subcutaneous fat and fat around the heart (*i.e.*, pericardial fat) and in the tongue, are independent risk factors for specific disorders (Fox *et al.* 2007, 2009; Rosito *et al.* 2008; Brennick *et al.* 2009; Mahabadi *et al.* 2009; Liu *et al.* 2010; Preis *et al.* 2010; Kim *et al.* 2014; Wang *et al.* 2020). For example, research has shown that pericardial fat is a particular risk factor for coronary artery calcification and coronary heart disease (Rosito *et al.* 2008; Mahabadi *et al.* 2009). Similarly, studies from our group have identified tongue fat as a key intermediate risk factor for OSA (Brennick *et al.* 2009; Kim *et al.* 2014) and the primary upper airway mediator of the relationship between weight loss and improvement in OSA severity (Wang *et al.* 2020).

Research also supports genetic influences on obesity-related traits. Multiple loci influence overall obesity (Locke *et al.* 2015). Moreover, emerging data support heritable components and genetic loci for specific fat distributions independent of general obesity (Fox *et al.* 2012; Schleinitz *et al.* 2014; Li and Qi 2019; Rask-Andersen *et al.* 2019; Pulit *et al.* 2019), including visceral and subcutaneous abdominal fat and pericardial fat. Genetic studies of tongue fat have not yet been conducted but are an important next step for identifying possible genetic risk factors for OSA, which is heritable (Gehrman *et al.* 2015). While genetic loci for obesity and fat distributions have been identified, there remains a large amount of unexplained heritability (Locke *et al.* 2015; Pulit *et al.* 2019). Ultimately, elucidating the specific genetic underpinnings of these intermediate traits is likely to improve our understanding of genetic and biological pathways affecting related complex disorders, including coronary heart disease and OSA.

One successful strategy for identifying relevant genes for many human diseases is to leverage mouse models. Recently, outbred mouse populations such as the Diversity Outbred (DO) mice (Churchill *et al.* 2012; Svenson *et al.* 2012; Gatti *et al.* 2014) have successfully identified narrow linkage peaks and significant gene-phenotype associations (Churchill *et al.* 2012; Svenson *et al.* 2012; Logan *et al.* 2013; Recla *et al.* 2014; Smallwood *et al.* 2014; French *et al.* 2015; Tyler *et al.* 2017; Gatti *et al.* 2018; Shorter *et al.* 2018; Yuan *et al.* 2018). Chosen based on community consensus, the eight founder mouse strains of the DO population include five classical inbred strains (A/J, C57BL/6J, 129S1/SvlmJ, NOD/LtJ, NZO/HILtJ) and three wild-derived strains representing different mouse sub-species (CAST/EiJ, PWK/PhJ, and WSB/EiJ). This diverse set of founder mice was used to generate the Collaborative Cross recombinant inbred strains via an eight-way funnel breeding scheme, with three outbreeding generations followed by repeated inbreeding (Churchill *et al.* 2004; Collaborative Cross Consortium 2012; Srivastava *et al.* 2017). Then, 144 independent Collaborative Cross lineages were used to seed the genetically and phenotypically heterogeneous DO mice, which are generated and maintained through randomized outbreeding (Churchill *et al.* 2012; Svenson *et al.* 2012; Gatti *et al.* 2014). As recently reviewed (Saul *et al.* 2019), combined experiments across founder strains, Collaborative Cross lines and Diversity Outbred mice represent a powerful approach to identify genetic associations with complex traits.

To leverage this approach, establishing the heritability of relevant endpoints in founder mice is crucial. Previous studies have shown genetic effects on specific fat distributions in mice (Li *et al.* 2006; Reed *et al.* 2006) and rats (Reed *et al.* 2011) based on the weights of fat depots. Available data within the eight founder strains of the Collaborative Cross and Diversity Outbred mice support heritability of body weight (Saul *et al.* 2019). While it has not been directly studied, given the inclusion of the very obese NZO/HILtJ strain (Taylor *et al.* 2001; Brennick *et al.* 2009) other fat distributions are likely to differ among founder strains and, thus, be heritable. To demonstrate this, the present study is the first to directly quantify specific fat distributions using novel three-dimensional magnetic resonance imaging (MRI) within each of the eight founder mouse strains, including the first to evaluate tongue fat across these strains.

The goals of this study were to describe a novel MRI-based phenotyping paradigm for quantifying different fat distributions and to calculate the heritability of each phenotype to understand which are most strongly influenced by genetics. We hypothesized that measures of fat distributions quantified using MRI would be significantly heritable, independent of general obesity (*e.g.*, body weight), in the founder strains of the Collaborative Cross and Diversity Outbred mice. Results represent a crucial first step for implementing quantitative genetic analyses using these new high-diversity mouse resources.

## Materials and methods

See Online Supplement for additional details.

### Animals and measurements

**Figure 1:**
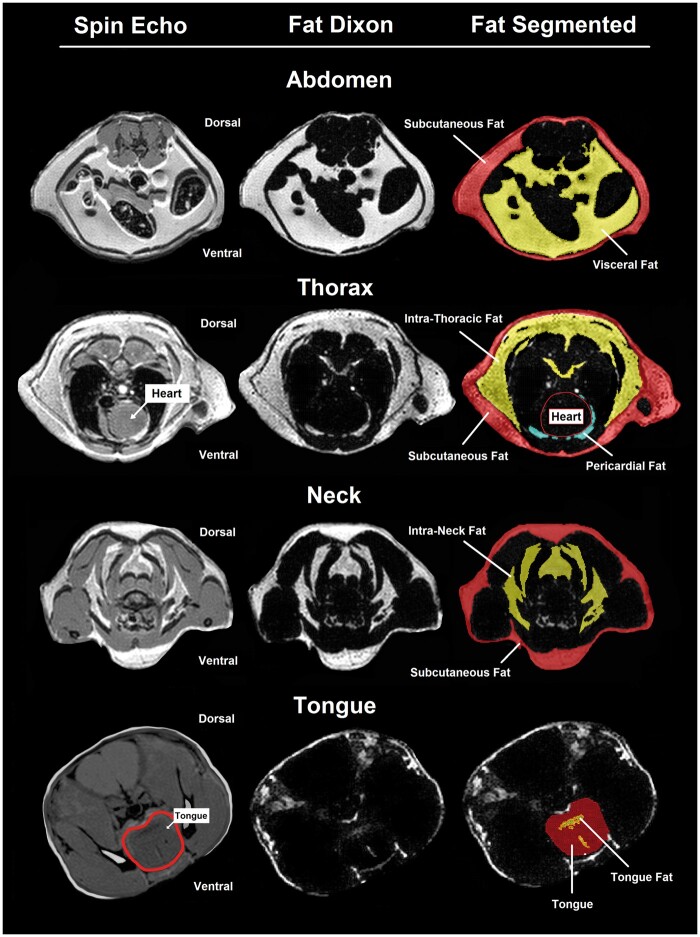
Illustration of MRI parameters in mice. Representative examples of axial T1 spin echo and Dixon scans, as well as segmentation of fat measurements, are shown for the abdomen, thorax and pericardium, neck and tongue. Subcutaneous fat is shown in pink/red, internal fat (*i.e.*, visceral abdominal, intra-neck, intrathoracic, and tongue) in yellow, and pericardial fat in blue.

This study was approved by the Institutional Animal Care and Use Committee (IACUC) of the University of Pennsylvania in accordance with the guidelines of the National Institutes of Health. Male mice from each of the five inbred [A/J (*n* = 12), C57BL/6J (*n* = 17), 129S1/SvlmJ (*n* = 12), NOD/LtJ (*n* = 14), NZO/HILtJ (*n* = 12)] and three wild-derived [CAST/EiJ (*n* = 14), WSB/EiJ (*n* = 15), PWK/PhJ (*n* = 12)] founder strains were delivered at age 6- to 8-weeks-old from the Jackson Laboratory to the small animal imaging facility at the University of Pennsylvania. Mice from the same strain were initially housed in cages with 5 mice and provided regular chow (Laboratory Rodent Diet 5001) and water *ad libitum* in a room with 12-h:12-h light:dark cycle (7 a.m.: 7 p.m.) with a lux level of approximately 100 lux at the bottom of the cage. Based on aggression, fighting or health issues, mice were moved to a cage with fewer mice or housed singly; thus, mice were ultimately housed in cages containing between 1 and 5 animals. At 6–7 months of age, mice were euthanized by cervical dislocation in conjunction with isoflurane anesthetic, weighed, and scanned with magnetic resonance Dixon and spin-echo imaging (see Figure 1).

### MRI and analysis

The MRI acquisition protocol is detailed in the Supplementary Materials. Images were analyzed using Amira 5.2.3 software (Amira, VSG, Burlington, MA). The program allowed segmentation of the tongue, neck, thorax, and abdomen using spin-echo images. These spin-echo images were overlaid onto Dixon images so that fat density could be effectively measured within the segmented areas using a threshold tool described previously by us (Brennick *et al.* 2009, 2014; Maislin *et al.* 2012; Kim *et al.* 2014; Wang *et al.* 2020). Total, subcutaneous and internal (*e.g.*, visceral [intra-abdominal], intrathoracic and intra-neck) fat volumes were quantified for the abdomen, thorax, and neck (see Figure 1). In addition, pericardial fat and tongue fat volumes were measured (see Figure 1).

### Statistical methods

We compared values among founder strains and calculated heritability for each phenotype. Values were compared among strains using analysis of variance (ANOVA). As all mice were exposed to the same environment, the proportion of phenotypic variability explained by strain provides an estimate of heritability. Specifically, heritability (h^2^) was calculated as the proportion of total variability attributable to differences in strains (*e.g.*, genetic variance) using a mixed-effects model with strain as a random effect to estimate the variance components [*e.g.*, h^2^ = (genetic variance)/(total variance)]. Since inbred mice are genetically identical within a strain, there are no dominance/interaction effects and this estimate represents narrow-sense heritability (Lariviere and Mogil 2010). A nonparametric 95% confidence interval (CI) around h^2^ was calculated as the 2.5th to 97.5th percentiles from 1,000 bootstrapped samples. A phenotype was considered significantly heritable if this CI did not overlap zero, indicating that underlying genetic architecture explained a significant (nonzero) proportion of variability. To understand whether heritability was driven by general obesity, we also calculated heritability using mixed-effects models including body weight (grams) as a covariate. Moreover, to evaluate whether observed heritability was attributable solely to the NZO/HILtJ strain, which is significantly more obese than other founder strains, analyses were repeated excluding data from NZO/HILtJ mice.

To reduce dimensionality and identify independent components explaining variability in fat distributions, we performed a principal components analysis. This analysis included eight fat volumes: subcutaneous and visceral abdominal fat, subcutaneous neck and intra-neck fat, subcutaneous thoracic and intrathoracic fat, pericardial fat, and tongue fat. Comparisons among founder strains and calculations of heritability were performed for the subset of principal components explaining >80% of variability in fat distributions, using the same methodology as described for individual fat distributions.

## Data availability

Additional details have been presented in the Supplementary Materials. Phenotype data on individual animals utilized in these analyses have been made available in Supplementary File S1. Detailed results, including strain-specific descriptive statistics, overall and pairwise comparisons, and heritability of observed and transformed outcomes are presented in Supplementary File S2 for analyses in all strains and in Supplementary File S3 after excluding the NZO/HILtJ strain. Supplemental Material available at figshare: https://doi.org/10.25387/g3.14188496.

## Results

### Phenotype distributions and heritability in founder strains

The following sections detail the observed phenotype distributions and heritability. Results were similar when using rank-based inverse normal transformations. Full results, including founder-specific means/standard deviations and medians/ranges, pairwise comparisons between strains, and heritability of observed and transformed outcomes are included in Supplementary File S2. Estimated heritability and descriptive comparisons of fat distributions excluding the NZO/HILtJ strain are presented in Supplementary File S3, and discussed briefly below.

**Figure 2:**
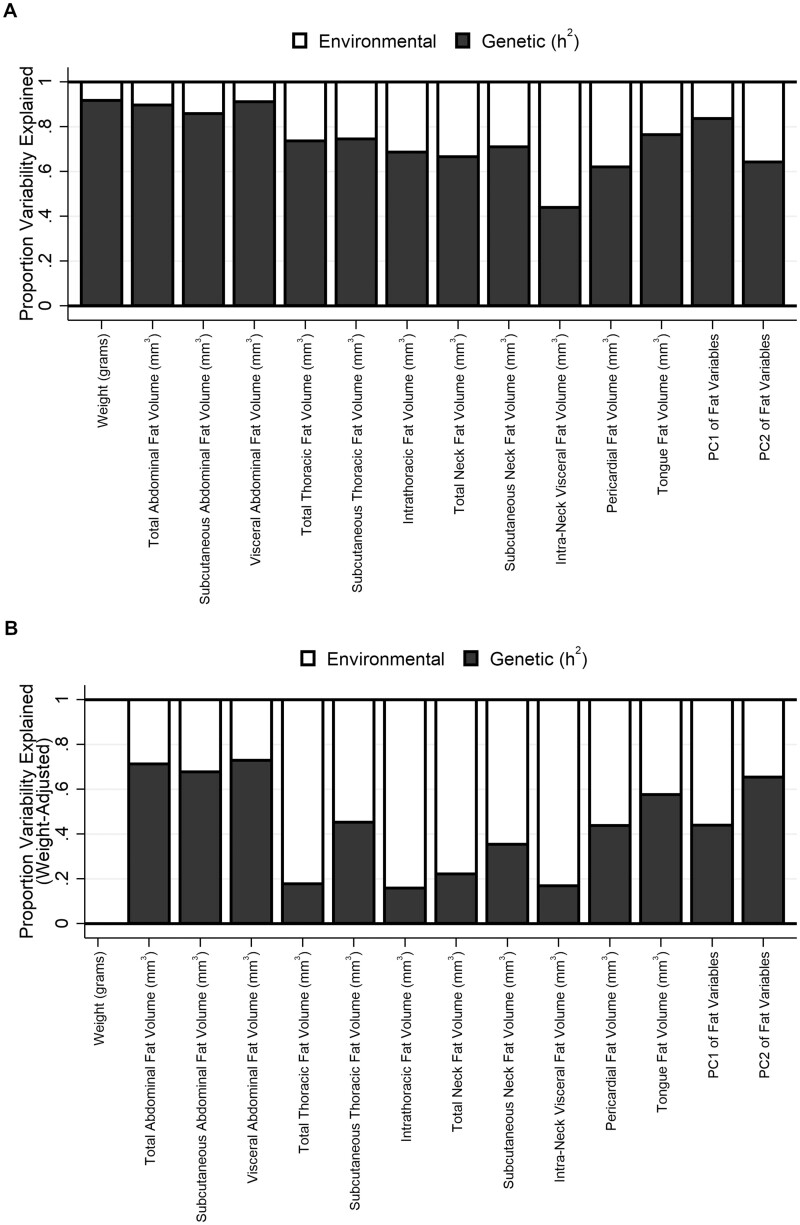
Heritability estimates across phenotypes. Estimates of the relative proportion of phenotypic variability explained by genetic factors (heritability; h^2^) and nongenetic factors (*e.g*., environmental) are shown across all phenotypes both (A) unadjusted and (B) controlling for body weight. Nearly all traits show high heritability in unadjusted analyses. While most estimates are reduced when adjusting for body weight, a number of traits maintain high heritability; the estimate for the second principal component (PC2) remains unchanged in adjusted analyses.

***Body weight:***Body weight was significantly different among founder strains (*p* = 4.5 × 10^−49^; Supplementary Figure S1). As expected, the NZO/HILtJ strain [55.3 ± 5.2 grams (g)] was significantly heavier than all other strains. Among the other founders, the classical inbred strains were heavier on average (28.1–30.6 g) than the wild-derived strains (18.4–25.2 g); nearly all pairwise differences were statistically significant (see also Supplementary File S2). Body weight was highly heritable (see Supplementary Figure S1 and Figure 2), with a heritability (95% CI) of 0.918 (0.888, 0.948).

***Subcutaneous and internal fat volumes in abdomen, thorax and neck:***Total, subcutaneous and internal [*e.g.*, visceral (intra-abdominal), intrathoracic and intra-neck] fat volumes were quantified for the abdomen, thorax and neck. Similar to body weight, all measures showed significant differences among founder strains, with significantly more fat in the NZO/HILtJ strain and often the least fat in the CAST/EiJ and WSB/EiJ strains (see Supplementary Figures S2–S4 and Supplementary File S2). Moreover, each fat distribution demonstrated significant and high heritability (see Figure 2).

Specifically, measures of abdominal total [h^2^ (95% CI) = 0.898 (0.850, 0.946)], subcutaneous [0.859 (0.802, 0.917)], and visceral [0.911 (0.868, 0.954)] fat were all highly heritable. Estimates were slightly reduced for total [0.713 (0.600, 0.826)], subcutaneous [0.678 (0.577, 0.778)], and visceral [0.729 (0.608, 0.851)] abdominal fat after controlling for body weight, but remained high (see Figure 2 and Supplementary Figure S2). For the thorax, measures of total [0.736 (0.602, 0.871)] and subcutaneous [0.746 (0.631, 0.861)] thoracic fat and intrathoracic fat [0.686 (0.504, 0.868)] were heritable. However, when controlling for body weight, only subcutaneous thoracic fat remained significant [0.453 (0.223, 0.684)], while estimates for intrathoracic and total thoracic fat were reduced and 95% CIs included 0% heritability (see Supplementary Figure S3). Similarly, in the neck we observed significant heritability for total [0.666 (0.487, 0.845)] and subcutaneous (0.710 [0.556, 0.865]) neck fat and for intra-neck fat (0.440 [0.254, 0.627]), but only subcutaneous neck fat remained significant when adjusting for body weight [0.354 (0.106, 0.601)] (see Supplementary Figure S4).

Therefore, results suggest heritability of fat distributions in the abdomen, thorax and neck, with the strongest evidence for effects independent of general obesity observed for both subcutaneous and visceral abdominal fat and for subcutaneous fat in the neck and thorax. Heritability of intrathoracic and intra-neck fat could be explained by genetic factors influencing overall body weight.

**Figure 3:**
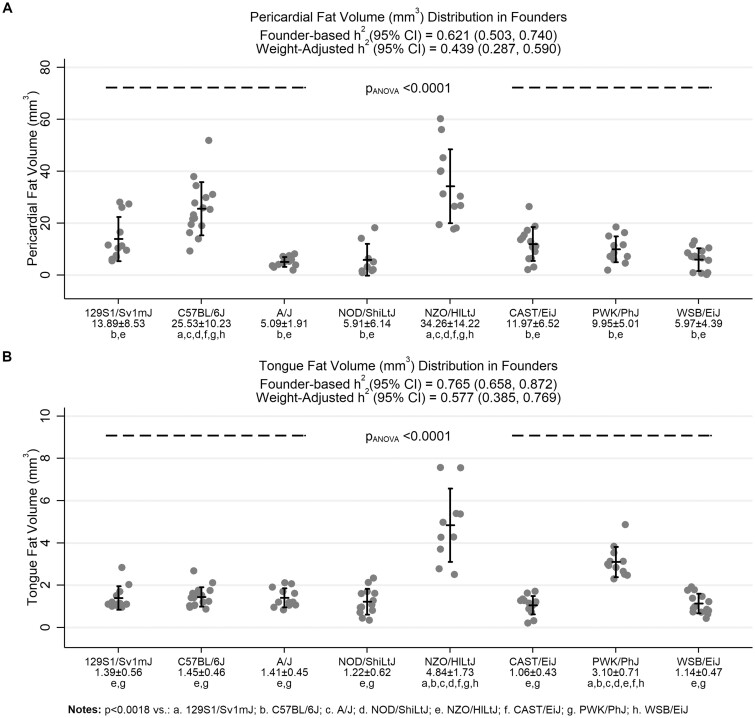
Comparison of pericardial fat and tongue fat volumes. The distribution of (A) pericardial fat and (B) tongue fat are shown across the eight founder strains. Vertical error bars represent the observed mean ± standard deviation. The NZO/HILtJ and C57BL/6J strains had significantly more pericardial fat than all other strains. We observed a significant heritability estimate of 0.621 (95% CI: 0.503, 0.740) for pericardial fat. This estimate remained significant after controlling for body weight (0.439 [0.287, 0.590]). For tongue fat, the NZO/HILtJ again demonstrated significantly more fat than other strains, but the PWK/PhJ strain, rather than C57BL/6J, also demonstrated significantly higher volume than all strains except NZO/HILtJ. Tongue fat volume had high heritability (0.765 [0.658, 0.872]), which remained significant when controlling for body weight (0.577 [0.385, 0.769]). Thus, both pericardial fat and tongue fat show significant heritability adjusting for body weight.

***Pericardial fat volume:***The amount of pericardial fat significantly differed across the founder strains (*p* = 3.3 × 10^−17^; see Figure 3A). Both the NZO/HILtJ (34.3 ± 14.2 mm^3^) and the C57BL/6J (25.5 ± 10.2 mm^3^) had significantly more pericardial fat than the other six founder strains, which each had pericardial fat volumes <14 mm^3^ on average. We observed significant heritability of 0.621 (0.503, 0.740). This estimate remained significant after controlling for body weight [0.439 (0.287, 0.590)], supporting evidence of genetic effects on pericardial fat independent of general obesity.

***Tongue fat volume:***As illustrated in Figure 3B, tongue fat volume significantly differed among strains (*p* = 1.2 × 10^−24^), with the NZO/HILtJ (4.84 ± 1.73 mm^3^) showing significantly more tongue fat than all other strains and the PWK/PhJ (3.10 ± 0.71 mm^3^) having less fat than the NZO/HILtJ, but significantly more tongue fat than the other six founder strains. Tongue fat volume had high heritability [0.765 (0.658, 0.872)], which remained significant when controlling for body weight [0.577 (0.385, 0.769)]. Thus, similar to pericardial fat, results support high heritability of tongue fat volume, with evidence of genetic effects independent of general obesity. Interestingly, while genetic factors in the NZO/HILtJ are likely to affect both these traits, the specific increases in pericardial and tongue fat volumes observed in the C57BL/6J and PWK/PhJ strains, respectively, suggest that genetic factors in these strains are also important.

### Principal components analysis of fat distributions

***Phenotype analysis:***As shown in Figure 4A, two principal components explained a total of 82.0% of the overall variability in fat distributions. The majority of variability (71.7%) was explained by the first component (PC1), which was strongly positively correlated with each of the individual fat distributions (see Table 1 and Figure 4B). Thus, PC1 captures the variability related to increased fat volumes overall. The second component (PC2) explained an additional 10.3% of variability in fat distributions; this component was most strongly positively correlated with intra-neck fat and pericardial fat, but had small, negative correlations with other fat volumes (Table 1 and Figure 4B). Thus, PC2 captures unique aspects of increased fat volume. Understanding the heritability of these two principal components can help to identify independent genetic effects.

**Figure:**
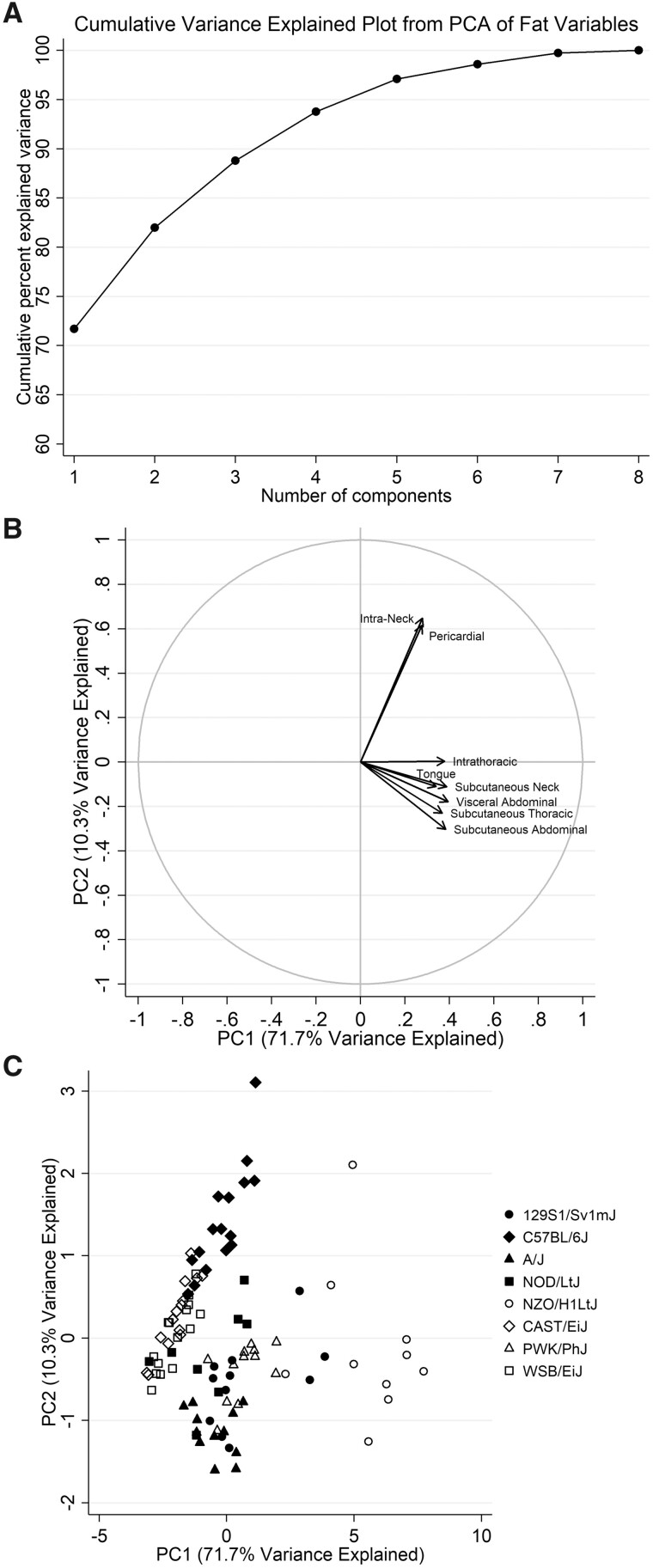
**Figure 4** Graphical summaries of principal components analysis. Graphs summarizing the results of the principal components analysis of individual fat distribution measures are shown, including (A) the cumulative variance explained, (B) a factor loading plot, and (C) a scatter plot. As shown in the plot of cumulative variance explained, the first two principal components explain >80% of the variability in fat distribution measures. The factor loading plot illustrates the relative correlations of each fat distribution with the two components, showing that all measures are positively correlated with PC1, whereas only intra-neck fat and pericardial fat are positively correlated with PC2. Finally, the scatter plot shows the values for individual mice of each strain.

**Table 1:** Correlations between principal components and individual fat distributions

Fat distribution	Correlations	
	PC1	PC2
Subcutaneous abdominal fat	**0.911**	**−0.275**
Visceral abdominal fat	**0.944**	**−**0.162
Subcutaneous thoracic fat	**0.880**	**−0.209**
Intrathoracic fat	**0.907**	0.003
Subcutaneous neck fat	**0.929**	**−**0.101
Intra-neck fat	**0.665**	**0.589**
Pericardial fat	**0.660**	**0.559**
Tongue fat	**0.813**	**−**0.100

Statistically significant (*P* < 0.05) correlations shown in bold.

PC, principal component.

**Figure 5:**
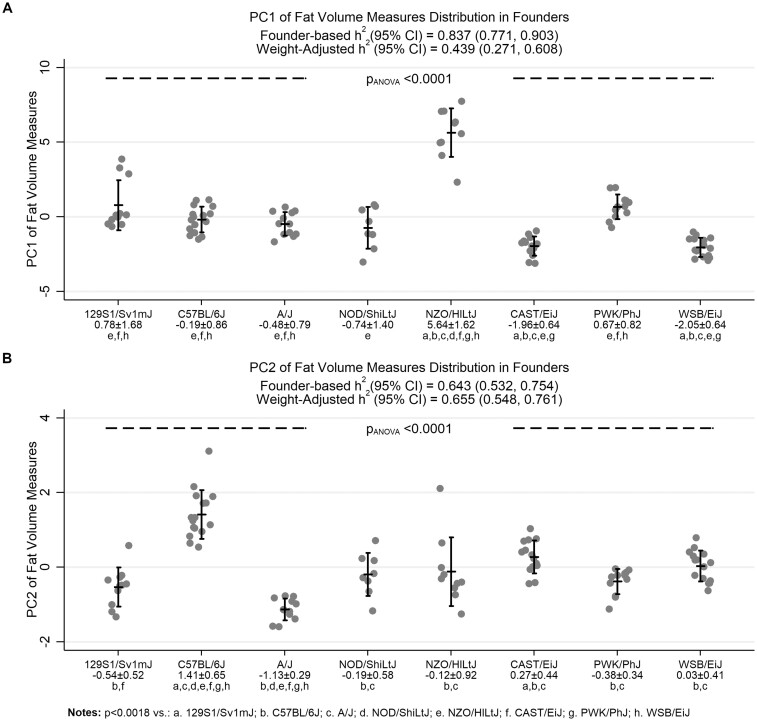
Comparison of principal components derived from individual fat distributions*.* Values of principal components are shown across the founder strains. Vertical error bars represent the observed mean ± standard deviation. Both components significantly differed among strains (*P* < 0.0001). The obese NZO/HILtJ strain had significantly higher values of PC1 compared to all other strains, reflecting the positive correlations between PC1 and all fat volume measures (see also Table 1). Results suggest this component is highly heritable, with an h^2^ (95% CI) of 0.837 (0.771, 0.903). Heritability is reduced by ∼50% when adjusting for body weight (0.439 [0.271, 0.608]). For PC2, which was most strongly correlated with intra-neck fat and pericardial fat volumes, the C57BL/6J mice have higher values than all other strains. This second component has high heritability (0.643 [0.532, 0.754]), which remains nearly identical after controlling for body weight (0.655 [0.548, 0.761]). Thus, results support a genetic influence on fat distributions, independent of general obesity.

***Distribution and heritability in founder strains:***Strong differences were observed for both principal components among strains (see Figure 4C and Figure 5). Given the positive correlations between PC1 and each fat distribution, the obese NZO/HILtJ strain had significantly higher values of this component than all other strains (Figure 5A). Results showed that PC1 is highly heritable [0.837 (0.771, 0.903)]. Heritability was reduced by nearly 50% when adjusting for body weight, but remained moderate [0.439 (0.271, 0.608)]. Conversely, PC2 captured specific increases in pericardial and intra-neck fat volumes. The C57BL/6J mice had significantly higher values of PC2 compared to all other strains (Figure 5B), supporting relatively increased pericardial and intra-neck fat within these mice. Results also showed that PC2 has high heritability, with an h^2^ (95% CI) of 0.643 (0.532, 0.754). This heritability estimate remained nearly identical after controlling for body weight [0.655 (0.548, 0.761)]; thus, there was a genetic influence on intra-neck and pericardial fat, independent of general obesity. Overall, results support the use of principal components in future studies to identify genetic effects on independent aspects of fat distribution, beyond those simply related to higher obesity in the NZO/HILtJ strain.

### Heritability estimates excluding the NZO/HILtJ strain

To better understand whether observed heritability of fat distributions was driven solely by genetic factors specific to the more obese NZO/HILtJ strain, we repeated heritability calculations excluding these mice (see Supplementary Figure S5 and Supplemental File S3). Across traits, unadjusted h^2^ estimates were comparable, but there was an average reduction in h^2^ of 14% after excluding the NZO/HILtJ (*P* = 0.0001; Supplementary Figure S5, A and B). While estimates were reduced on average, all measures remained significantly heritable; h^2^ estimates ranged from 43 to 73% with none of the 95% CIs including 0% heritability (see Supplementary File S3). When comparing h^2^ adjusted for body weight, estimates were again comparable across traits, but weight-adjusted h^2^ values were 7% higher on average in analyses excluding the NZO/HILtJ strain (*P* = 0.005; Supplementary Figure S5, C and D). Ultimately, these results are consistent with the idea that observed heritability of fat distributions is not simply driven by the NZO/HILtJ mice, and that genetic determinants after excluding the NZO/HILtJ mice are less driven by general obesity. While overall variability is changed by excluding the NZO/HILtJ strain, results show that fat distributions are influenced by underlying genetic factors even among the less obese founder strains.

## Discussion

Our data demonstrate the distributions and heritability of body weight and specific fat measurements, including subcutaneous and internal fat in the abdomen, thorax and neck, pericardial fat, and tongue fat. For each measure, we observed significant differences among the eight founder strains of the Collaborative Cross and Diversity Outbred mouse populations, along with significant heritability. The majority of heritability estimates remained moderately high after controlling for body weight, including heritability of subcutaneous fat in the abdomen, neck and thorax, visceral abdominal fat, pericardial fat and tongue fat, supporting genetic influences on these fat distributions independent of general obesity. Results of principal components analysis on fat volume measurements suggest that two components explain >80% of variability; the first is strongly related to overall increased fat, while the second was specific to increased intra-neck fat and pericardial fat. Both components demonstrated significant heritability, with the heritability of the second principal component remaining unchanged when controlling for body weight. Therefore, results support genetic influences on both overall obesity and specific fat distributions, laying the foundation for future studies leveraging Collaborative Cross and/or Diversity Outbred mice to identify new genes affecting variability in these traits.

### Evidence of genetic influence on obesity and fat distributions in humans and mice

Multiple genetic factors contribute to determining overall obesity and fat distributions, and the results of the present study are consistent with prior studies in humans and mice. As reviewed by Li and Qi (2019), heritability estimates range from 40 to 70% for BMI, 30–45% for measures of regional fat distribution such as waist circumference and waist-to-hip ratio (controlling for BMI), and are 36 and 57% for visceral and subcutaneous adipose tissue, respectively, based on computed tomography. Moreover, as discussed in a recent review on utilizing mouse resources for studying complex traits, available data in the eight founder strains studied here support heritability of body weight (Saul *et al.* 2019). To our knowledge, no published studies have examined heritability of individual fat deposits or utilized novel MRI-based phenotyping approaches within each of these founder strains. Thus, our results provide important and new information in this area.

Beyond demonstrating heritability, studies have identified specific genetic factors affecting overall obesity and fat distributions. Using F_2_ hybrid mice, Reed *et al.* performed a linkage analysis of both absolute and relative (*e.g.*, adjusted for body weight) retroperitoneal and gonadal adipose tissue weights (Reed *et al.* 2006). This analysis identified 67 suggestive associations across phenotypes. Sex-specific genetic associations were also identified; while fat distributions were more heritable in female than male mice, linkage peaks specific to males were also shown (Reed *et al.* 2006). More sophisticated analysis approaches in mice (Li *et al.* 2006) and a previous study in rats (Reed *et al.* 2011) also support specific genetic effects on fat distributions based on depot weights.

In humans, multiple studies have found individual genes affecting BMI and fat distributions (Fox *et al.* 2012; Schleinitz *et al.* 2014; Locke *et al.* 2015; Li and Qi 2019; Pulit *et al.* 2019; Rask-Andersen *et al.* 2019). In a meta-analysis of 322,154 individuals of European ancestry and 17,072 individuals of non-European ancestry, Locke *et al.* (2015) identified 97 loci associated with BMI, 56 of which were novel. Five loci harbored multiple, independent genetic effects on BMI, highlighting the complex genetic architecture underlying obesity. While a large number of loci were identified, the significantly associated single nucleotide polymorphisms (SNPs) only accounted for 2.7% of the variability in BMI. All common SNPs (regardless of significance) were estimated to account for as much as 21% of the variance (Locke *et al.* 2015), suggesting a large amount of unexplained heritability.

Regarding specific fat distributions, both targeted and large-scale analyses have shown unique genetic associations. A genome-wide association study including patients of European and non-European ancestry identified a significant relationship between a variant in the *TRIB2* gene (rs10198628) and pericardial fat (Fox *et al.* 2012). This variant was not associated with BMI or visceral abdominal fat (Fox *et al.* 2012). Furthermore, two recent large-scale studies leveraging the UK Biobank (Rask-Andersen *et al.* 2019) or a meta-analysis (Pulit *et al.* 2019) have identified associated loci and sex-specific genetic effects on fat distributions. Specifically, Rask-Andersen *et al.* (2019) studied the predicted proportion of body fat in the arms, legs and trunk using segmental bio-electrical impedance analysis (an estimate of total adipose tissue based on electrical impedance through the body), weight, age, and height among 362,499 individuals in the UK Biobank. Ninety-eight independent associations were identified, including 29 novel ones. Results supported stronger associations in females than males for 37 associated genetic variants. Similarly, in a meta-analysis evaluating genetic associations with waist-to-hip ratio adjusted for BMI in 694,649 individuals, Pulit *et al.* (2019) identified 463 independent associations at 346 genetic loci. Among the individual SNPs identified, 105 showed evidence of sex-specific associations, with 97 (92.4%) having stronger associations in females. As observed for BMI (Locke *et al.* 2015), despite the large number of identified loci, variants associated in this meta-analysis only accounted for 3.9% of phenotypic variability, while overall SNP-based heritability was estimated to be 17.4% (Pulit *et al.* 2019).

Thus, our observation of genetic effects both related to and independent of overall obesity is consistent with results from large-scale studies in humans. However, results in humans indicate a large proportion of unexplained heritability. While less frequent variants with larger effects or epistasis could explain some of this missing heritability (Eichler *et al.* 2010), more accurate phenotyping methods in humans or animals may be required to uncover these genetic associations. The MRI-based phenotyping described here could represent one such approach, particularly when combined with animal models that allow tighter control of environmental factors (*e.g*., diet) that may confound association analyses in humans. In particular, studying these phenotypes within the heterogeneous DO mice is likely to identify new and robust genetic associations.

### Implications for complex diseases related to specific fat distributions

Particular distributions of excess fat capture risk for specific conditions better than overall obesity (Cornier *et al.* 2011). Thus, elucidating the specific genetic factors influencing fat distributions using mice will not only provide mechanistic insights for these traits, but will also further understanding of the genetic and biological pathways affecting related complex disorders.

In particular, visceral abdominal fat is a risk factor for metabolic disease, as well as triglycerides, HDL cholesterol, and abnormal blood glucose (Fox *et al.* 2007, 2009; Liu *et al.* 2010; Preis *et al.* 2010). Both pericardial and intrathoracic fat are associated with increased risk of coronary artery and/or abdominal aortic calcification, independent of visceral abdominal fat and other cardiovascular risk factors (Rosito *et al.* 2008). Pericardial fat is also an independent risk factor for coronary artery disease (Rosito *et al.* 2008; Fox *et al.* 2009; Mahabadi *et al.* 2009). Genetics of cardiovascular and coronary artery disease have been reviewed extensively (Kathiresan and Srivastava 2012; Khera and Kathiresan 2017), with recent articles describing the importance of both polygenic risk scores and clinical or lifestyle factors (Khera *et al.* 2016; Knowles and Ashley 2018; Said *et al.* 2018). Animal models, such as the Diversity Outbred and Collaborative Cross mice, are likely to continue to play an important role in determining causality for these genetic associations identified in humans (Khera and Kathiresan 2017; Saul *et al.* 2019).

In addition to cardiovascular diseases, the results of the present study have implications for genetics of OSA, a disease with increasing prevalence that is associated with numerous comorbidities (Punjabi 2008; Lim and Pack 2017). OSA is heritable, but validated genetic variants remain elusive (Gehrman *et al.* 2015). Recent GWAS have discovered some genes and variants associated with OSA (Cade *et al.* 2016; Chen *et al.* 2018; Farias Tempaku *et al.* 2020; Strausz *et al.* 2020), but these findings need to be replicated. Importantly, tongue fat is directly linked to OSA (Kim *et al.* 2014; Wang *et al.* 2020), including regional differences in tongue fat distribution (Kim *et al.* 2014) and evidence that reduced tongue fat mediates associations between weight loss and improved OSA severity (Wang *et al.* 2020). Currently, there are no reported studies examining the genetic determinants of tongue fat, which our study shows is heritable in mice. It is likely that future studies on the genetic predictors of tongue fat in Diversity Outbred and Collaborative Cross mice, as well as related studies of other relevant anatomy quantifiable with MRI-based methods, will lead to novel genetic associations relevant to OSA.

### Future applications for quantitative genetic analysis in mice

Establishing the heritability of fat distributions within the founder strains of the Collaborative Cross and Diversity Outbred mice demonstrates the likely utility of leveraging these high-diversity mouse models to identify specific genes affecting these traits. Thus, our results serve as the crucial foundation for future studies using these mouse resources. As detailed elsewhere (Churchill *et al.* 2012; Svenson *et al.* 2012; Gatti *et al.* 2014), DO mice are an outbred mouse population seeded by 144 Collaborative Cross strains and maintained through random outbreeding. This breeding strategy results in high phenotypic and genetic heterogeneity among mice that more closely resembles diversity seen in human populations. Using established analytic approaches (Gatti *et al.* 2014; Saul *et al.* 2019), this heterogeneity can be leveraged to identify small linkage peaks (containing only a few genes) and increases the likelihood of large genetic effects on heritable phenotypes. Once an association has been discovered in DO mice, subsequent studies in a smaller number of Collaborative Cross mice (Churchill *et al.* 2004; Collaborative Cross Consortium 2012; Srivastava *et al.* 2017) carrying the implicated founder alleles at specific locations can then be leveraged to validate associations and conduct more targeted, mechanistic studies.

In addition to providing broad justification for studying these traits in Diversity Outbred and Collaborative Cross mice, results of the present analyses can provide insights into expected strain-specific genetic effects. For example, significantly greater pericardial fat was observed in the C57BL/6J and NZO/HILtJ strains and greater tongue fat volume was observed in the PWK/PhJ and NZO/HILtJ strains. Thus, when studying Collaborative Cross or Diversity Outbred mice, we may expect to find independent genetic effects for pericardial and tongue fat volume both related to general obesity (likely due to genetic factors inherited from the NZO/HILtJ) and independent of general obesity (likely driven by genetic factors inherited from C57BL/6J or PWK/PhJ). Consistent with the idea of multiple strain-specific genetic factors, traits studied here remained significantly heritable when excluding the NZO/HILtJ mice from calculations.

Overall, given the heritability estimates demonstrated in our study, future analyses in the Diversity Outbred and Collaborative Cross mice are likely to identify novel gene variants and/or complex genetic effects influencing the distribution of fat in the upper airway and abdomen, as evidenced by a number of prior studies on other heritable phenotypes (Churchill *et al.* 2012; Svenson *et al.* 2012; Logan *et al.* 2013; Recla *et al.* 2014; Smallwood *et al.* 2014; French *et al.* 2015; Tyler *et al.* 2017; Gatti *et al.* 2018; Shorter *et al.* 2018; Yuan *et al.* 2018).

### Strengths and limitations

This study has a number of strengths. Mice are an ideal animal in which to identify genes for specific traits related to human disease. We performed quantitative imaging of fat distributions using novel three-dimensional MRI in mice, based on similar techniques from our previous studies (Brennick *et al.* 2009, 2014; Maislin *et al.* 2012; Kim *et al.* 2014; Wang *et al.* 2020). Not only do these techniques have advantages over prior studies in mice that largely relied on weighing regional fat depots, but also the ability to apply similar phenotyping methods in mice and humans facilitates more efficient translation. The inclusion of mice from each of the eight founder strains of the Collaborative Cross and Diversity Outbred populations to quantify heritability is another strength, as are the robust analytic methods applied.

There are also limitations to this study. Foremost, our study only included male mice. Prior research in humans and mice suggests differences in fat distributions between males and females (Reed *et al.* 2006; Karastergiou *et al.* 2012). Similarly, there are sex-related differences in the genetic associations with fat distributions, with evidence of stronger genetic effects in females (Reed *et al.* 2006; Karastergiou *et al.* 2012; Li and Qi 2019; Pulit *et al.* 2019; Rask-Andersen *et al.* 2019). Thus, there is need to conduct analyses in both male and female mice. Our analyses in males represent the first step towards understanding the genetic influences of MRI-based fat distributions in mice. Based on prior data we would expect these traits to be equally or more heritable in female mice. Thus, our data likely represent a lower bound on the overall heritability. In addition, while the heritability estimates presented here evaluate the phenotypic variability explained by differences in strain, the lack of dominance or interaction effects across founder strains results in an estimate of narrow-sense, rather than broad-sense heritability (Falconer and Mackay 1996; Lariviere and Mogil 2010). Relatedly, estimates of heritability derived in founder mice attribute differences between strains solely to genetics; it is possible that unknown nongenetic effects could influence differences and bias h^2^ estimates, although this is unlikely given our careful control of environment. Within a given strain, we generally observed expected variability in body weight that is consistent with data from other studies (Evsikova and Svenson 2009; Bogue *et al.* 2015, 2020; Kollmus *et al.* 2020). A number of nongenetic factors may have contributed to this variability, including social hierarchy among mice in the same cage or development of underlying conditions affecting weight, such as diabetes in a subset of the NZO/HILtJ (Kleinert *et al.* 2018) or NOD/LtJ (Bao *et al.* 2002) mice. While we were not able to adjust for these factors in our heritability calculations, any increase in within-strain variance caused by nongenetic factors would be expected to decrease, rather than increase, estimates of heritability. Finally, to understand whether genetic influences on fat distributions were independent of general obesity, we performed analyses adjusted for body weight. While this is a commonly used measure of overall adiposity, body weight is also influenced by fat free mass (Heymsfield *et al.* 2014). Adjusting for fat free mass could provide more robust estimates of fat-specific heritability; however, a reliable measure of fat free mass was not available in our data.

### Conclusions

Our findings demonstrate that fat distributions are heritable within the eight founder strains of the Collaborative Cross and Diversity Outbred mice. Thus, the data suggest a strong potential for gene discovery using MRI to directly quantify measures of fat distributions in these high-diversity mouse resources (Saul *et al.* 2019). Our results confirm high heritability of body weight, and provide new data on the heritability of volumes of subcutaneous and internal fat in the abdomen, thorax and neck, pericardial fat volume, and tongue fat volume in these founder mice. Highlighting the distinct genetic architecture influencing these traits, heritability estimates of most individual fat distributions remained moderately high after controlling for general obesity (body weight). Results of principal components analysis on fat distributions further emphasized this point; the second component was specific to intra-neck fat and pericardial fat and heritability of this component remained unchanged when controlling for body weight. Ultimately, these results provide the foundation for future discovery analyses of genes determining specific fat distributions using Collaborative Cross and Diversity Outbred mice. This, in turn, can shed light on the biological mechanisms through which these fat distributions exert specific effects on complex traits in humans, including cardiovascular disease and OSA.
